# Oral colonization by *Candida* species and associated factors in HIV-infected patients in Ahvaz, southwest Iran

**DOI:** 10.4178/epih.e2020033

**Published:** 2020-05-24

**Authors:** Elham Aboualigalehdari, Maryam Tahmasebi Birgani, Mahnaz Fatahinia, Mehran Hosseinzadeh

**Affiliations:** 1 Department of Medical Mycology, School of Medicine, Ahvaz Jundishapur University of Medical Sciences, Ahvaz, Iran; 2 Department of Medical Genetics, School of Medicine, Ahvaz Jundishapur University of Medical Sciences, Ahvaz, Iran; 3 Infectious and Tropical Diseases Research Center, Health Research Institute, School of Medicine, Ahvaz Jundishapur University of Medical Sciences, Ahvaz, Iran; 4 Thalassemia and Hemoglobinopathy Research Center, Health Research Institute, Ahvaz Jundishapur University of Medical Sciences, Ahvaz, Iran

**Keywords:** Candidiasis, Oral, Species, Colonization, Human immunodeficiency virus, Iran

## Abstract

**OBJECTIVES:**

Oropharyngeal candidiasis is one of the most common opportunistic fungal infections among human immunodeficiency virus (HIV)-infected individuals. The most common cause is *Candida albicans*, followed by non-*albicans Candida*. This study aimed to identify colonized *Candida* species in HIV-infected patients from Ahvaz, Iran. Additionally, the relationships between immunity-related factors, lifestyle, and colonization of *Candida* spp. were studied.

**METHODS:**

Oral swabs were taken from 201 HIV-positive patients referred for consultations at the Behavioral Modification Center. Oral *Candida* colonization was detected using culture-based and molecular assays. Data were assessed by descriptive statistics and analyzed to investigate the correlation between *Candida* colonization and various factors, including the CD4^+^ cell count and viral load.

**RESULTS:**

It was found that 43.8% of patients were positive for *Candida*. The most common species was *C. albicans* (48.0%), followed by non-*albicans Candida* isolates, including *C. dubliniensis*, *C. glabrata*, *C. tropicalis*, *C. parapsilosis*, *C. guilliermondii*, *C. kefyr*, and *C. krusei*. Colonization of *Candida* spp. in patients was associated with a CD4 count ≤200 cells/mm^3^ (odds ratio [OR], 4.62; p<0.05), history of shared injections (OR, 6.96; p<0.001), and sex (OR, 3.59; p<0.05).

**CONCLUSIONS:**

The results of this study showed that *C. albicans* was the dominant pathogen. The risk factors for colonization of *Candida* spp. were a CD4 count ≤ 200/mm^3^ , a history of shared injections, and sex. Other factors with potential relationships include viral load, age, and opportunistic infections, but further investigations are needed.

## INTRODUCTION

Oropharyngeal candidiasis (OPC) is an opportunistic fungal infection that affects the oral mucosa. Although *Candida albicans* is the most common causative agent of OPC, non-*albicans Candida* species have also been reported to be responsible for OPC, which is mostly observed in patients undergoing chemotherapy and in transplant recipients. OPC is also one of the most frequent infections in human immunodeficiency virus (HIV)-infected patients, as it has been observed in more than 90% of HIV patients. Remarkably, OPC has been detected in early stages of HIV before treatment, as well as in advanced stages of acquired immune deficiency syndrome (AIDS) [1,2].

OPC is mostly diagnosed through its clinical manifestations, which include a burning sensation, buccal pain, taste changes, and pseudomembranous, erythematous, and angular cheilitis [3,4]. To treat OPC, it is necessary to identify the causative species because a majority of infections contain mixed *Candida* spp. Both *C. albicans* and non-*albicans Candida* spp. may be affected by acquired and intrinsic antifungal resistance, which could influence the management of the disease and contribute to the spread of candidiasis to esophageal tissue, malnutrition, and interference with drug absorption. Consequently, the identification of the causative species is necessary and the diagnosis should be made using both culture and molecular methods [5-7]. Oral candidiasis is a prognostic indicator in HIV patients, as well as a predisposing factor for fungal infections such as candidemia and esophageal candidiasis. Dental prostheses, smoking, and hormonal, nutritional, immunological, and salivary changes predispose HIV patients to oral candidiasis [3,8,9].

The current study aimed to evaluate the colonization of *Candida* spp. in HIV-positive patients, to determine the distribution of *Candida* spp., and to identify the relationships of *Candida* spp. colonization with a CD4 count ≤ 200 cells/mm^3^, viral load, history of shared injections, sex, age, intravenous drug use, underlying disease (hepatitis C, tuberculosis [TB], hepatitis B, *Pneumocystis carinii* pneumonia [PCP]), having a safe and secure sexual relationship, the use of antiretroviral medication, and the use of prophylactic medication (for PCP and TB).

## MATERIALS AND METHODS

### Study subjects

HIV-infected outpatients aged 6-81 years (n=201) were referred for consultations to the Behavioral Disease Center of Ahvaz Jundishapur University of Medical Sciences, Ahvaz, Iran from December 2018 to April 2019 for viral load and CD4^+^ T-cell count measurements. HIV positivity was the sole inclusion and exclusion criterion for sampling.

The subjects were interviewed about their medical history and information was extracted from their medical records on sex, age, intravenous drug use, underlying diseases (hepatitis C, TB, hepatitis B, and PCP), whether they had a safe and secure sexual relationship, antiretroviral medication use, and history of using prophylaxis drugs (for PCP and TB).

### Initial identification of yeasts

Samples from the oral cavity of HIV-positive participants were collected with a sterile cotton swab, seeded onto CHROMagar *Candida* plates (CHROMagar, Pioneer, Paris, France), and incubated aerobically at 37°C for 48-72 hours. Colonies with similar morphology and color on CHROMagar plates were counted and the result was expressed in colony-forming units per milliliter (CFU/mL) after 48-72 hours of incubation at 37°C on CHROMagar plates. Patients with ≥ 10 CFU/mL were considered to be colonized [10].

Patients who were asymptomatic for oral candidiasis with ≥ 10 CFU/mL were defined as having been colonized by *Candida*, but asymptomatic patients with < 10 CFU/mL were considered noncolonized. The next step was the purification of isolates, which were cultured on Sabouraud Dextrose agar (Lifoilchem, Roseto degli Abruzzi, Italy). The initial identification of isolates to the species level was performed by classical and standard methods such as germ tube test, growth at 45°C, and production of chlamydoconidia and hyphae on corn meal agar (Lifoilchem) with Tween 80.

### Molecular identification of yeasts

Firstly, the genomic DNA was directly extracted from purified colonies in sterile distilled water by boiling [6,11]. Polymerase chain reaction (PCR) was performed by sequencing an internal transcribed spacer (ITS1-5.8S rDNA-ITS2) region and using universal fungal primers (Table 1). The PCR amplification conditions consisted of an initial denaturation at 95°C for 5 minutes, followed by 35 cycles at 95°C for 35 seconds, 58°C for 30 seconds, 72°C for 1 minute, and a final extension at 72°C for 10 minutes. All PCR tests had a negative (no template) control and a positive (sequence template) control. Finally, to determine the species, the positive specimens were identified by restriction fragment length polymorphisms [12]. Each of the amplified PCR products was digested using MspI (Hpa III; Thermo Fisher Scientific, Waltham, MA, USA) restriction enzymes and incubated at 37°C for 1-16 hours. The digested products were run on 2% agarose gel and stained with 0.5 μg/mL ethidium bromide. Since the MspI enzyme is not able to differentiate *C. albicans* from *C. dubliniensis* or *C. albicans* from *C. dubliniensis*, isolates suspected to be *C. albicans* or *C. dubliniensis* were distinguished by duplex PCR using primers targeting sequences in the ITS-1 and ITS-2 regions of the rDNA (Table 1) [13]. The PCR reaction started at 95°C for 5 minutes, followed by 35 cycles of 95°C for 30 seconds, 60°C for 45 seconds, 72°C for 30 seconds, and a final extension at 72°C for 5 minutes.

### Statistical analysis

Data were assessed using descriptive statistics (frequency, percentage, mean, standard deviation). Associations between categorical variables were analyzed using the chi-square test or Fisher exact test. Variables with p-value< 0.2 in the univariate analysis were included in the multiple logistic regression analysis model. Multicollinearity was checked, and all variables had a variance inflation factor well below 5. The Hosmer-Lemeshow test was used to evaluate the goodness-of-fit for multiple logistic regression. The Kolmogorov-Smirnov test was used to assess the normality of the distribution of continuous variables, and variables that did not have a normal distribution were compared using the Mann–Whitney test. All analyses were performed using R version 3.5.2 (https://cran.r-project.org/bin/windows/base/old/3.5.2/) and SPSS version 22 (IBM Corp., Armonk, NY, USA). A p-value< 0.05 was set as the threshold for statistical significance.

### Ethics statement

This cross-sectional study received ethical approval from the research deputy of Jundishapur University of Medical Sciences, Ahvaz, Iran (code #1397/893).

## RESULTS

The current study analyzed 201 HIV-infected patients, including 115 male and 86 female patients aged 6-81 years. Patients’ sociodemographic characteristics are summarized in Table 2. The colonized (≥ 10 CFU/mL) and non-colonized groups (< 10 CFU/mL) accounted for 43.8% and 56.2% of the sample, respectively. No statistically significant differences were found between the 2 groups in terms of TB prophylaxis, PCP, antiretroviral therapy, blood transfusion, mother-to-child transmission, addiction history, homosexuality, education, occupational exposure, and hepatitis B or hepatitis C virus infection, although people with male sex, a history of drug injection, TB, a history of shared injections, and a CD4 count ≤ 200/mm^3^ were more likely to be colonized (p<0.05).

The age distribution and *Candida* load in the colonized group were significantly different from those of the non-colonized group, although the viral load was similar in the 2 groups (Table 3). The p-value of the Hosmer–Lemeshow test was 0.387, indicating a goodness-of-fit data. The factors associated with colonization were subjected to multiple logistic regression analysis. Sex (odds ratio [OR], 3.59; 95% confidence interval [CI], 1.23 to 10.46; p<0.05), a history of shared injections (OR, 6.96; 95% CI, 2.46 to 19.65; p<0.001), and CD4 count (OR, 4.62; 95% CI, 1.43 to 14.90; p<0.05) showed significant differences between the colonized and noncolonized groups (Table 4).

From the study population, 127 strains of *Candida* were isolated in 88 colonized patients. *C. albicans* was the most common strain isolated, accounting for 48.0% of isolates (61/127), while non-*albicans Candida* strains were found in the remaining 52.0% of isolates (66/127). The most common species among the non-*albicans Candida* isolates were *C. dubliniensis* and *C. glabrata*, with prevalence rates of 22.8% (n= 29) and 14.1% (n= 18), respectively. The distribution of the isolated species is shown in Table 5.

In 32 patients (36.4%), more than 1 *Candida* spp. was isolated. The cocolonization distribution is shown in Table 6.

## DISCUSSION

The aim of this study was to obtain epidemiological data on the colonization of different *Candida* species in HIV-infected patients in Ahvaz, Iran. HIV patients have a high incidence of *Candida* colonization and are more susceptible to OPC [14,15].

In our study, the rate of *Candida* colonization in patients with HIV infection was 43.8%, which is similar to those reported in other studies conducted in China (49.5%), Brazil (50.4%), and Taiwan (51.4%) [3,16]. However, a very broad range of *Candida* colonization rates among HIV-infected patients (11-96%) has been reported in other studies [10]. The prevalence of *Candida* spp. colonization may vary based on factors such as sampling methods, diagnostic techniques, and geographical and ethnic differences [10,17]. Colonization ranges of 16-56% have been reported using swab mouth sampling [10].

In a review study by Patil et al. [18] in 2018, the most common strain isolated from HIV patients with OPC was *C. albicans* (37.2-95.2%), followed by *C. glabrata* (0.99-23.00%). In our study, *C. albicans* was the most common oral yeast in HIV-infected patients with a frequency of 69.3% [19-22]. On one hand, *C. dubliniensis* has been reported to be the second most common and dominant non-*albicans Candida* spp. in some studies, followed by *C. glabrata* [2,22-24]; on the other hand, other studies have identified *C. glabrata* as the most common non-*albicans Candida* spp. [19,25, 26]. In recent years, we have witnessed an increase in *C. dubliniensis* as the most prevalent species after *C. albicans* even in healthy individuals without HIV [27,28]. The absence of molecular techniques in the past when species were identified using traditional methods may explain this phenomenon, because *C. dubliniensis* is similar to *C. albicans* and has wrongly been reported as *C. albicans*.

The increase in *C. dubliniensis* among HIV patients is concerning because exposure to fluconazole may aggravate fungal adhesion to the oral epithelium. Since HIV-infected patients receive fluconazole to prevent this adhesion [27,29], there is a risk of increasing oropharyngeal candidiasis with *C. dubliniensis* and other non-*albicans Candida* spp., as well as resistance to fluconazole, leading to prolonged treatment and higher morbidity and mortality rates due to OPC [5,30-32].

In our study of 88 colonized patients, 59 (67.0%) had only 1 type of *Candida* spp. and the remaining patients (32.9%) had a combination of 2 or 3 yeast species. It can be clinically challenging when susceptible and less susceptible or resistant species to an antifungal medication coexist (i.e., coinfection), such as *C. glabrata* and *C. krusei*, which are less susceptible to fluconazole and voriconazole and were present in 14.1% and 0.7% of *Candida* carriers in this study, respectively. In contrast, *C. parapsilosis* and *C. tropicalis* (3.9% and 6.2% of *Candida* carriers in this study, respectively) have a similar sensitivity to that of *C. albicans* [18].

These observations underscore the importance of species assessment using *Candida* CHROMagar medium, which is a low-cost and convenient method of species identification, especially for samples involving more than 1 species [33]. The factors potentially related to oral colonization by *Candida* spp. in HIV-infected individuals were evaluated in this study. Through multiple logistic regression analysis, one of the risk factors for *Candida* colonization was identified as a CD4 count ≤ 200/mm^3^ (OR, 4.62; 95% CI, 1.43 to 14.90; p<0.05). Therefore, in this study, the oral cavity of HIV-infected individuals with a low CD4 count was approximately 5 times more likely to be colonized by *Candida* spp., implying that the environment in the oral cavity of these patients was likely 5 times more immunosuppressive. Therefore, *Candida* colonization could be considered as a proxy marker of immunosuppression based on the findings of this study, with potential for use in circumstances where it is not possible to measure CD4 counts. Our results are consistent with those of other studies in this respect [32,34-38]. According to the observations of this study, shared injections increased the risk of *Candida* colonization in HIV patients by approximately 7-fold compared to other HIV patients (OR, 6.96; 95% CI, 2.46 to 19.65; p<0.001), which aligns with the findings of other studies [32,39,40]. Furthermore, the colonization rate of males was approximately 4 times higher than that of females (OR, 3.59; 95% CI, 1.23 to 10.46; p<0.05), which may reflect the higher prevalence of HIV in males [41,42]. Other conditions that may occur, depending on the status of the patient’s immune system, include opportunistic infections such as TB. An analysis using the chi-square test showed a significant relationship between TB and oral *Candida* colonization (p<0.05). Consistent with other studies [43], oral candidiasis can be a useful clinical tool for screening HIV patients for TB, and we recommend that when an HIV patient with oral candidiasis is referred to a clinic, he or she should be checked for TB and receive prophylaxis for TB [44]. Viral load is another host-related factor, but despite experiments indicating that HIV load reduces immunity on mucosal surfaces and increases the secretion of acid protease from *C. albicans*, the Mann-Whitney test showed that our results were not statistically significant; this finding is in agreement with some studies, but not with others [10,45,46]. A possible explanation for this phenomenon might be that insufficient information was available about patients’ viral load and that the test was performed among existing patients; therefore, the insufficient data in this study might not have had sufficient statistical power to show these differences.

Our results showed the rate of *Candida* colonization and *Candida* spp. abundance and diversity in HIV-infected patients in Ahvaz, Iran. *C. albicans* was dominant on the oral mucosal surfaces of these patients, and *C. dubliniensis* was the most common yeast among non-albicans*Candida* spp. A CD4 count of ≤200/mm^3^ had a strong association with *Candida* colonization, confirming that the cellular immune response plays a vital role in protecting against OPC and that an impaired immune response could be a risk factor. A number of factors, such as viral load and opportunistic infections, require further investigation in larger populations of patients. Therefore, there is a need for further research on this issue in Iran to better clarify these findings.

## Figures and Tables

**Table 1.** List of primers used in this study

Primers	Reference
ITS1 5´-TCC-GTA-GGT-GAA-CCTGCG-G-3´	[9]
ITS4 5´-TCC-TCC-GCT-TAT-TGA-TAT-GC-3´	
CALF 5´-TGGTAAGGCGGGATCGCTT-3´	[13]
CALR 5´-GGTCAAAGTTTGAAGATATAC-3´	
CDUF 5´-AA ACTTGTCACGAGATTATTTTT-3´	[13]
CDUR 5´-AAA GTTTGAAGAATAAAATGGC-3	

**Table 2.** The frequency of socio-demographic characteristics of the study subjects

Variables	Colonized (n=88)	Non-colonized (n=113)	p-value^1^
Sex			
Male	63 (54.8)	52 (45.2)	<0.001
Female	25 (29.1)	61 (70.9)	
Drug injection			
True	47 (57.3)	35 (42.7)	0.001
False	41 (34.5)	78 (65.5)	
TB			
Yes	15 (68.2)	7 (31.8)	0.015
No	73 (40.8)	106 (59.2)	
TB prophylaxis			
Yes	6 (30.0)	14 (70.0)	0.191
No	82 (45.3)	99 (54.7)	
PCP			
Positive	12 (63.2)	7 (36.8)	0.074
Negative	76 (41.8)	106 (58.2)	
ARV			
Yes	85 (42.9)	113 (57.1)	0.082
No	3 (100)	0 (0.0)	
Blood transfusion			
True	1 (100)	0 (0.0)	0.438
False	87 (43.5)	113 (56.5)	
Mother-to-child transmission			
True	3 (60.0)	2 (40.0)	0.655
False	85 (43.4)	111 (56.6)	
Addiction history			
Yes	51 (49.5)	52 (50.5)	0.093
No	37 (37.8)	61 (62.2)	
Education			
No education	11 (57.9)	8 (42.1)	0.193^2^
Elementary education	75 (41.7)	105 (58.2)	
Higher education	2 (100)	0 (0.0)	
Homosexuality			
True	5 (71.4)	2 (28.6)	0.244
False	83 (42.8)	111 (57.2)	
HCV Ag			
Positive	31 (50.0)	31 (50.0)	0.235
Negative	57 (41.0)	82 (59.0)	
HBV Ag			
Positive	4 (50.0)	4 (50.0)	0.732
Negative	84 (43.5)	109 (56.5)	
Occupational exposure			
True	1 (50.0)	1 (50.0)	1.000
False	87 (43.7)	112 (56.3)	
Shared injection history			
Yes	37 (72.5)	14 (27.5)	<0.001
No	51 (34.0)	99 (66.0)	
CD4 count (cells/mm^3^)			
≤200	20 (80.0)	5 (20.0)	<0.001
>200	68 (38.6)	108 (61.4)	

Values are presented as number (%). TB, tuberculosis; PCP, *Pneumocystis carinii* pneumonia; ARV, antiretroviral; HCV, hepatitis C virus; HBV, hepatitis B virus; Ag, antigen.

1 Chi-square test or Fisher exact test.

2 Tests were performed after pooling the last 2 groups (elementary education, higher education) in order to eliminate small expected frequencies.

**Table 3.** Characteristics of the study population

Variables	Colonized		Non-colonized		p-value^1^
	n	Mean±SD	n	Mean±SD	
Age	88	40.61±12.55	113	37.15±8.18	0.005
Viral load	36	802,637.71±2,544,107.27	27	3,150,032.340±11,343,051.38	0.064
*Candida* load	88	5,787.51±23,256.64	58	3.50±2.12	<0.001

SD, standard deviation.

1 Mann-Whitney test.

**Table 4.** Prevalence rates of oral colonization by *Candida* species

Variables	OR (95% CI)	p-value
Sex (ref: female)	3.59 (1.23, 10.46)	0.019
Age	1.01 (0.98, 1.04)	0.376
Drug injection (ref: false)	0.78 (0.22, 2.68)	0.697
TB (ref: no)	1.75 (0.55, 5.57)	0.341
TB prophylaxis (ref: no)	0.65 (0.21, 2.03)	0.466
PCP (ref: negative)	1.10 (0.31, 3.89)	0.872
Addiction history (ref: no)	0.28 (0.07, 1.03)	0.056
Education (ref: yes)	1.98 (0.64, 6.17)	0.234
Shared injection history (ref: no)	6.96 (2.46, 19.65)	<0.001
CD4 (ref: >200)	4.62 (1.43, 14.90)	0.010
Hosmer–Lemeshow test^1^: χ^2^=8.49, p=0.387		

OR, odds ratio; CI, confidence interval; TB, tuberculosis; PCP, *Pneumocystis carinii* pneumonia; ref, reference.

1 The Hosmer–Lemeshow test is a statistical test for goodness-of-fit for logistic regression models. It is used frequently in risk prediction models.

**Table 5.** Distribution of *Candida* strains (n=127) isolated from colonized patients (n=88)

Species	Isolation rate by species, n (%)	Isolation rate by patients, %
*Candida albicans*	61 (48.0)	69.3
*Candida dubliniensis*	29 (22.8)	32.9
*Candida glabrata*	18 (14.1)	20.4
*Candida tropicalis*	8 (6.2)	9.1
*Candida parapsilosis*	5 (3.9)	5.6
*Candida guilliermondii*	3 (2.3)	3.4
*Candida kefyr*	3 (2.3)	3.4
*Candida krusei*	1 (0.7)	1.1

**Table 6.** Distribution of co-colonization with different yeast species in 88 colonized patients

Mixed colonization of species	Patients, n (%)
*C. albicans*+*C. dubliniensis*	6 (6.8)
*C. albicans*+*C. glabrata*	5 (5.6)
*C. albicans*+*C. dublinensis*+*C. glabrata*	4 (4.5)
*C. dubiliansis*+*C. glabrata*	4 (4.5)
*C. albicans*+*C. tropicalis*	2 (2.2)
*C. albicans*+* C. guilliermondii*	2 (2.2)
*C. albicans*+*C. dubilinensis*+*C. kefyr*	1 (1.1)
*C. albicans*+*C. glabrata*+*C. tropicalis*	1 (1.1)
*C. dubiliansis*+*C. parapsilosis*	1 (1.1)
*C. dubilinensis*+*C. tropicalis*	1 (1.1)
*C. glabrata*+*C. tropicalis*	1 (1.1)
*C. dubilinensis*+*C. kefyr*	1 (1.1)
